# Comparison of commonly used methods in random effects meta-analysis: application to preclinical data in drug discovery research

**DOI:** 10.1136/bmjos-2020-100074

**Published:** 2021-02-25

**Authors:** Ezgi Tanriver-Ayder, Christel Faes, Tom van de Casteele, Sarah K McCann, Malcolm R Macleod

**Affiliations:** 1 Centre for Clinical Brain Sciences, Edinburgh Medical School, The University of Edinburgh, Edinburgh, Scotland, UK; 2 Translational Medicine and Early Development Statistics, Janssen Pharmaceutica, Beerse, Antwerpen, Belgium; 3 Data Science Institute (DSI), Interuniversity Institute for Biostatistics and Statistical Bioinformatics (I-BioStat), Hasselt University, Hasselt, Limburg, Belgium; 4 QUEST Center for Transforming Biomedical Research, Berlin Institute of Health (BIH), Charité–Universitätsmedizin Berlin, Berlin, Germany

**Keywords:** preclinical animal studies, aggregate data meta-analysis, between-study heterogeneity, Bayesian analysis, meta-regression

## Abstract

**Background:**

Meta-analysis of preclinical data is used to evaluate the consistency of findings and to inform the design and conduct of future studies. Unlike clinical meta-analysis, preclinical data often involve many heterogeneous studies reporting outcomes from a small number of animals. Here, we review the methodological challenges in preclinical meta-analysis in estimating and explaining heterogeneity in treatment effects.

**Methods:**

Assuming aggregate-level data, we focus on two topics: (1) estimation of heterogeneity using commonly used methods in preclinical meta-analysis: method of moments (DerSimonian and Laird; DL), maximum likelihood (restricted maximum likelihood; REML) and Bayesian approach; (2) comparison of univariate versus multivariable meta-regression for adjusting estimated treatment effects for heterogeneity. Using data from a systematic review on the efficacy of interleukin-1 receptor antagonist in animals with stroke, we compare these methods, and explore the impact of multiple covariates on the treatment effects.

**Results:**

We observed that the three methods for estimating heterogeneity yielded similar estimates for the overall effect, but different estimates for between-study variability. The proportion of heterogeneity explained by a covariate is estimated larger using REML and the Bayesian method as compared with DL. Multivariable meta-regression explains more heterogeneity than univariate meta-regression.

**Conclusions:**

Our findings highlight the importance of careful selection of the estimation method and the use of multivariable meta-regression to explain heterogeneity. There was no difference between REML and the Bayesian method and both methods are recommended over DL. Multiple meta-regression is worthwhile to explain heterogeneity by more than one variable, reducing more variability than any univariate models and increasing the explained proportion of heterogeneity.

**Strengths and limitations of this study**

- Although general comparison of heterogeneity estimation methods for random effects meta-analysis exists based on clinical data, to our knowledge, this is the first analysis to compare the most commonly used approaches for estimating heterogeneity in preclinical data, which consist of a large number of small studies with highly heterogeneous observed treatment effects, unlike clinical data. Our findings are relevant to all meta-analyses with similar data structures, which combine data from a large number of small studies.
- This is a summary and application of the most common methods for estimating and quantifying heterogeneity in meta-analysis of preclinical data and serves as an example for preclinical researchers wanting to conduct multiple meta-regression.
- Restricted maximum likelihood and Bayesian methods should be preferred over DerSimonian and Laird for estimating heterogeneity in meta-analysis especially when there is high heterogeneity in the observed treatment effects across studies. Multivariable meta-regression explains substantially more heterogeneity than univariate meta-regression and it should be preferred to investigate the relationship between the treatment effect and multiple study design and characteristic variables.
- The methods used for estimating and quantifying heterogeneity in meta-analysis of aggregate data assume normality which may not always be the case in preclinical data given the small within-study sample sizes.

- In this study, we compare the methods in the setting of a typical preclinical meta-analysis of aggregate data. However, no simulation has been done to investigate the generalisability of the results.

## Background

Preclinical animal experiments are deployed to deepen our understanding of human disease mechanisms and to develop candidate treatments for humans. Despite this, the number of treatments reaching the clinic is still limited. It is explicit that there are limitations to the effectiveness of translation from preclinical to clinical research as it currently exists. Given the large volume of preclinical research, it is challenging to summarise the available evidence for treatment effects before launching clinical trials. Systematic review and meta-analysis are tools that can tackle some of these problems in translational research by providing a summary of research findings and evaluation of their internal and external validity.

Systematic review is a structured method to obtain all data related to a specific research question, and these data may then be analysed using meta-analysis. Meta-analysis is commonly used in many fields to derive summary information from the data reported in these research papers. Meta-analysis in preclinical animal studies attempts to compare and combine results across related studies to understand differences between studies with different characteristics. The observed treatment effect obtained in each study is an estimate of the true treatment effect, with variability due to chance and due to differences in study design, conduct and methodology, variation due to these differences being known as heterogeneity.^1^ Investigating possible causes of this variation between studies helps us to understand which factors might influence the effect size and inform how we might design future experiments. Several methods for obtaining an overall treatment estimate and accounting for the between-study variance have been discussed, including both Bayesian and frequentist approaches.^2–4^ The two most commonly used methods to provide an overall summary of the treatment effect are the fixed effects and random effects models.^5^ Subgroup analysis and meta-regression are the most commonly used approaches to explain heterogeneity. Although the literature provides some discussion on the usefulness of these methods based on clinical data, there are few examples specific to preclinical data. An important characteristic of preclinical data identified in systematic review is that it usually comprises a large number of studies which each reports outcomes from a small number of animals; and with larger heterogeneity between studies as compared with clinical data.^6^ Given differences in selection and inclusion for preclinical compared with clinical studies, we understand that heterogeneity in clinical trial populations (due to age, sex, and so on) is contained within individual studies; whereas in preclinical research that heterogeneity lies between studies. In view of this, we aim to explore and illustrate the differences of the most commonly used estimation techniques for heterogeneity and the overall effect estimate, and compare simple and multiple meta-regression analyses from aggregate-level study data to explain sources of heterogeneity present among studies.

We review and illustrate current statistical approaches for preclinical meta-analysis and meta-regression, and the most common techniques for estimating between-study variability, including method of moments, likelihood and Bayesian approaches. To motivate our paper, we introduce the study quality and design characteristics of systematic review data related to the efficacy of interleukin-1 receptor antagonist (IL-1RA) in animal models of stroke. Lastly, we discuss limitations and make recommendations for the use of these methods.

## Methods

### Data: systematic review of IL-1RA in animal models of stroke

We reassess a published meta-analysis describing the efficacy of IL-1RA in animals exposed to focal cerebral ischaemia (modelling human stroke) using the summary data extracted as part of the systematic review conducted by the Collaborative Approach to Meta Analysis and Review of Animal Data from Experimental Studies - CAMARADES group.^7^ IL-1RA is used clinically to treat rheumatoid arthritis but has also been investigated as a therapy for stroke. The difference in the effects of IL-1RA compared with a control group exposed to vehicle or no treatment in in vivo animal studies of focal cerebral ischaemia is the outcome of interest. The primary endpoint used in the meta-analysis was infarct volume. The secondary endpoints were neurobehavioural scores and mortality.

Twenty-five publications were included in the systematic review. Infarct volume was measured in 76 experiments involving 1283 animals, neurobehavioural score in 33 experiments from 473 animals and mortality in 10 experiments from 227 animals.

For infarct volume and neurobehavioural scores, information consists of means and SDs together with the number of animals present in each intervention group and study, which are used to calculate the effect size on a normalised mean difference (NMD) scale. Details on how to calculate the effect size based on NMD can be found here.^6^ Information on mortality is binary and is summarised as the number of animals out of the total number of animals that died in each group and study, allowing us to obtain the effect size based on log OR and its SE.

Both study quality and design characteristics were investigated. Note that only variables with sufficient information at all levels of the variable were considered. For some variables, levels with fewer than 10 observations were combined into one category. Tables 1 and 2 provide an overview of the variables, summarising the study qualities and characteristics.

**Table 1:** Description of study quality variables provided in the data sets

Variables	Data type	Description
Control of temperature during stroke induction	Categorical	True, false
Random allocation to group	Categorical	True, false
Blinded induction of ischaemia	Categorical	True, false
Blinded assessment of outcome	Categorical	True, false
Anaesthetic without marked intrinsic neuroprotective activity	Categorical	True, false
Use of comorbid animals	Categorical	True, false
Sample size calculation	Categorical	True, false
Compliance with animal welfare regulations	Categorical	True, false
Statement of potential conflicts of interest	Categorical	True, false
Monitoring of physiological variables during stroke induction	Categorical	True, false
Prespecified inclusion-exclusion criteria	Categorical	True, false
Reporting of excluded animals	Categorical	True, false
Injury confirmed by laser Doppler	Categorical	True, false

**Table 2:** Description of study characteristic variables provided in the data sets

Variables	Data type	Description
Nature of IL-1RA delivered	Categorical	Vector, protein, transgenic, bone marrow cells
Time of first drug administration	Continuous	Time after induction drug is administrated (min)
Time to outcome measure	Continuous	Time it takes to assessment of outcome (min)
Cumulative drug dose	Continuous	Dose provided in first 24 hours of administration
Route of drug delivery	Categorical	Intracerebroventricular, intravenous, subcutaneously, other
Number of drug administration	Categorical	Multiple, other
Type of ischaemia	Categorical	Temporary, other
Method of ischaemic occlusion	Categorical	Electrocoagulation, intraluminal filament, other
Anaesthetic used	Categorical	Isoflurane, halothane, other
Method of infarct measurement	Categorical	Cresyl violet, TTC, other
Species	Categorical	Rat, mouse
Strain	Categorical	Sprague Dawley, Wistar, C57BL/6, other
Mode of delivery	Categorical	Central, peripheral
Correction for oedema	Categorical	Corrected, other
Comorbidity	Categorical	None, other
Published pre-2009	Categorical	True, false

IL-1RA, interleukin-1 receptor antagonist; TTC, triphenyltetrazolium chloride.

### Statistical models for meta-analysis

In this section, we review the statistical techniques used for meta-analysis performed on an outcome measure (effect size), recorded in each study. The general fixed effects approach is introduced in Fixed Effects Approach section. For situations where heterogeneity is expected to be present in the data, we let the summary treatment effect parameter change from one study to another by making use of random effects model.^5^ This approach is presented in Random Effects Approach section.

#### Fixed effects approach

Given a collection of *k* independent studies, each study *i* compares an experimental treatment group (E) with a control group (C). The response variable of interest, effect size, is obtained for each study. The effect size, represented by parameter *θ_i_*, is the difference in effect of the experimental treatment in comparison to the control in study *i*. This *θ_i_* can be equal to the log OR for binary mortality data or the NMD for the continuous outcomes infarct volume and neurobehavioural scores, which links the effect size in treatment group to control group with reference to the outcome observed in a normal unlesioned (‘sham’) animal not exposed to focal cerebral ischaemia.^6^ Let $\hat{\theta_{i}}$ denote an estimate of $\theta_{i}$ from study *i*. The general parametric fixed effects model is written as^8^:

(1) $${\hat{\theta }}_{i}=\theta +\epsilon_{i}$$

where *i*=1, *…*, *k* with $\epsilon_{i}∼N\left(0,\xi_{i}^{2}\right)$. Generally, it is assumed that study variabilities $\xi_{i}^{2}$ are known. Using the least squares approach, common fixed effects treatment difference can be extracted. The weighted least squares estimator is then given as:

(2) $${\hat{\theta }}_{F}=\frac{\sum_{i=1}^{k}{\hat{\theta }}_{i}w_{i}}{\sum_{i=1}^{k}w_{i}}$$

with $w_{i}=1/\xi_{i}^{2}$ representing the weight of each study. The SE of the fixed effects estimate is

(3) $$se\left({\hat{\theta }}_{F}\right)=\sqrt{\frac{1}{\sum_{i=1}^{k}w_{i}}}$$

with a 95% CI for *θ* equal to $\hat{\theta }\pm 1.96se({\hat{\theta }}_{F})$.^9^

##### Test of heterogeneity of effect sizes across studies

Testing whether the effect sizes do not vary between studies can be done using the same F-test in analysis of variance to test that study means are equal.^10^ Although several methods for testing the assumption that the effect sizes are homogeneous across studies have been introduced,^11^ the most common way of estimating the extent of heterogeneity and testing its significance is by means of the *Q* statistic which measures the deviation of each individual study effect size estimate from the overall effect size^8 12^:

(4) $$Q=\sum_{i=1}^{k}w_{i}{\left({\hat{\theta }}_{i}-{\hat{\theta }}_{F}\right)}^{2}$$

where *w_i_*=1*/ξ_i_*^2^. This *Q* statistic follows a χ^2^ distribution with *k−1* df under the hypothesis of homogeneity.^13 14^ When *Q* is larger than the critical value, then this implies that the effect sizes vary more from one another than would be expected based on sampling variability solely. A disadvantage of this statistic is that the test may have low power if the meta-analysis consists of small number of studies and may have high power if there are large numbers of studies although the amount of variability in the true treatment effects is negligible.^11 15^ I^2^ estimate, which describes the percentage of heterogeneity that is due to between-study differences, has become a common statistic used by researchers as an index of heterogeneity.^15^

(5) $$\begin{array}{c} I^{2}=100\%\left(\frac{Q-df}{Q}\right) \end{array}$$

The I^2^ statistic can also be described as the percentage ratio of heterogeneity (between-study variability) to total variability (between-study and within-study variability).

I^2^ ranges from 0% to 100%, with larger values implying increasing heterogeneity.^15 16^ However, it is important to note that I^2^ is a percentage, not an absolute value.^17^ Therefore, it does not inform us on how much variation there is in effect sizes. The main advantage of I^2^ is that it can be used to compare meta-analyses of different sample sizes, different types of studies and outcome data.^15^

#### Random effects approach

Under the fixed effects model, it is assumed that there is one true effect size that underlies all the studies included in our analysis. Accordingly, it is assumed that the differences in observed effects are only due to sampling error. Under the random effects model, we permit the true effect to vary from one study to another. The effect sizes observed in the studies are believed to be a random sample of these effect sizes.^18^ In the random effects model, the treatment difference parameters in *k* studies (*θ*_1_,*…*,*θ_k_*) are believed to be a random sample of independent observations from N(μ, *τ*^2^). The general random effects model is presented as follows:

(6) $${\hat{\theta }}_{i}=\mu +b_{i}+\epsilon_{i}$$

where *b_i_* are normally distributed random effects reflecting study to study variation with $\epsilon_{i}∼N\left(0,\xi_{i}^{2}\right)$. As before, it is assumed that $\xi_{i}^{2}$ is known and the two sources of variability, $b_{i}$ and $\epsilon_{i}$, are assumed to be independent.^3^ This implies that the observed effect sizes are assumed to be normally distributed, given by ${\hat{\theta }}_{i}=N\left(\mu ,\;\xi_{i}^{2}+\tau^{2}\right)$.

Hence, the true effect for the $i^{th}$ study is centred around the overall effect μ. Between-study heterogeneity (*τ*^2^) needs to be estimated from the data as it is usually unknown. Assuming ${\left(w_{i}^{*}\right)}^{-1}=(\xi_{i}^{2}+\tau^{2})$ represents the true variance of $\hat{\theta_{i}}$, the maximum likelihood estimate of μ can be shown to be:

(7) $${\hat{\theta }}_{R}=\frac{\sum_{i=1}^{k}{\hat{\theta }}_{i}w_{i}^{*}}{\sum_{i=1}^{k}w_{i}^{*}}$$

with SE

(8) $$se\left({\hat{\theta }}_{R}\right)=\sqrt{\frac{1}{\sum_{i=1}^{k}w_{i}^{*}}}$$

95% CI for μ is equal to ${\hat{\theta }}_{R}\pm 1.96se\left({\hat{\theta }}_{R}\right)$.^9^

It is important to realise that the fixed effects model is a special case of the random effects model, where *τ*^2^ is set to be 0. The adjusted weights *w_i_*^∗^ are observed to be close to the original weights *w_i_* when the *τ*^2^ is small. Accordingly, in such a case, the results from SE, CI and the overall effect size estimate calculated using the random effects approach will be identical to the ones obtained using the fixed effects modelling approach. When *τ*^2^ is large, the SE and CI will be larger than that of the fixed effects model. Three approaches are commonly used to estimate *τ*^2^: the DerSimonian and Laird (DL) method, the restricted maximum likelihood (REML) method and the Bayesian method. Although there are alternative frequentist approaches^19^ to reflect the current state of knowledge, in this paper we chose to focus on DL and REML.

##### DL method

DL, a moment-based estimator, is the oldest and has historically been the most commonly implemented method in preclinical meta-analysis as it is calculated directly rather than requiring an iterative procedure.^19 20^ Hence, it is the default approach in many software programmes for meta-analysis. The DL estimator is derived by comparing the observed value of *Q* and its expectation. When the test statistic *Q* is smaller than its df, the DL estimate shows no evidence for the presence of between-study heterogeneity, and a fixed effects analysis is acceptable. However, default use of the DL method has often been challenged in clinical meta-analysis, as it may underestimate the between-study heterogeneity, leading to smaller CIs for the mean effect, specifically when the between-study heterogeneity is large, as in preclinical meta-analysis settings.^21 22^ Although the decision to apply a fixed versus random effects model should be driven by the inference model, some researchers make this choice on the basis of a statistical test of heterogeneity, which usually has low power.^23^ Furthermore, rather than relying on an overall statistical test to detect the presence of heterogeneity, it may be more important to quantify the extent of heterogeneity and explain its sources.^24^ Hence, the use of statistical tests of heterogeneity is not encouraged and rather than relying on an estimate made by a traditionally conservative estimator of heterogeneity, the decision to proceed with fixed or random effects model should be based on the research question and model assumptions. More extensive discussion can be found in the literature.^5 18 23–25^

##### Likelihood approach

Alternatively, REML, a more computationally demanding iterative likelihood-based approach, is the second most popularly used method in practice. This is since it is easily implemented in the software packages for meta-analysis and is known to yield unbiased estimates for variance components.^22^ For a meta-analysis with *k* independent studies, the likelihood is obtained by the product of individual study likelihoods. Maximum likelihood estimates of *τ*^2^ and μ may be obtained through an iterative procedure. Maximum likelihood method is known to underestimate *τ*^2^ as it neglects the information used in μ estimation. Hence, REML approach can be used instead by redefining log likelihood only in terms of the variance parameters.^2^

In addition to these most commonly used techniques focused on this paper, other methods to estimate the value of *τ*^2^ include Paule-Mandel, Hedges estimator and Hunter-Schmidt estimator, which are method of moments estimators; Sidik-Jonkman estimator, which is a model error variance estimator; and empirical Bayes estimator, which is a Bayes estimator.^14^

##### Bayesian approach

Here, we also consider a Bayesian approach to the random effects meta-analysis model. In this framework, all parameters are assumed as random variables in Bayesian methodology. A prior distribution is specified for each parameter and reflects our prior knowledge on the parameters. The posterior distribution combines the prior with the likelihood derived from the data. In meta-analysis, we have two main parameters, namely: the effect size estimate μ and the heterogeneity estimate *τ*^2^. The impact of the choice of the prior distribution on the posterior distribution for μ depends on the amount of information provided by the studies in the meta-analysis. Moreover, the prior distribution chosen is especially important for *τ*^2^ when there are only a small number of studies included in the analysis.^9^ Based on the prior information and the knowledge of the researcher, non-informative (vague) or informative priors can be specified. Vague priors express our a priori uncertainty on the subject and permit the data to determine the posterior while an informative prior represents high certainty in the range of parameter values and dominates the posterior, requiring more data to shift that credibility.^26^ In many meta-analyses, a vague prior distribution is chosen especially for the overall effect μ such that the inference on the primary interest is derived based on the observed data alone.^27^ A wide normal distribution is a particularly convenient and commonly used prior in meta-analysis with most common effect measures such as mean differences, standardised mean differences and (log) odds, risk and HRs.^27 28^ As previously indicated, since the prior distribution of the heterogeneity estimate *τ*^2^ can often be influential, it should be selected with particular care. Uniform distribution, half normal and half Cauchy are convenient prior distributions for the heterogeneity parameter.^26^ Alternatively, inverse gamma prior distribution is recommended for heterogeneity as it is known to result in better convergence and bias.^26 29^ However, it should be noted that the performance of estimators may vary depending on the type of effect measure of interest. Hence, sensitivity analysis is recommended to assess the impact of different prior specifications for *τ*^2^. More in-depth discussion on the choice of vague priors for the heterogeneity parameter can be found in the literature.^28–30^ For our analysis to reflect our ignorance about the parameters, a vague prior is assumed.^29^ When the likelihood is normally distributed as in our data, the common conjugate prior for the mean is specified to be a normal distribution while for variance it is assumed to follow an inverse gamma distribution.

(9) $$\mu ∼N(\theta_{0},\sigma_{0}^{2})$$

$$\tau^{2}∼IG\left(\alpha ,\lambda \right)$$

Inferences are obtained using Markov chain Monte Carlo Gibbs sampler implemented via OpenBUGS software.^31^ To ensure adequate convergence, results are conventionally obtained using two chains of 10 000 iterations with a burn-in period of 1000 iterations.

##### Fixed effects versus random effects model

Decisions on model selection should be made based on a judgement of whether the studies share a common effect. Although results of a statistical test for heterogeneity supply further information about the variability, a decision should not be made purely on the basis of the p value obtained, and these results should not inform decisions on whether to present an overall fixed effects or random effects estimate of the treatment difference. Such a decision should be determined in advance and described in the study protocol.

Results from a fixed effects analysis cannot be generalised beyond the studies for which data were used in the meta-analysis. Thus, overall estimation of the treatment effect obtained using a fixed effects approach presents a summary of the results from the particular sample of animals contributing to data retrieved from each study. A random effects approach makes results generalisable to the whole population of studies from which the studies used for the meta-analysis were (assumed to be) drawn.^9^ This is reflected in the width of the CI, especially if there is a considerable amount of variability among the studies.

### Meta-regression

Heterogeneity in systematic review data may be completely random or may occur because of differences between studies that are associated with differences in effect size.^32^ This can be investigated by meta-regression models, where estimates of treatment differences are taken as dependent variables and study-level characteristics as independent variables. This approach may face two challenges. First, a large number of independent variables can be identified as potential sources of heterogeneity, and depending on the number of studies included in the meta-analyses, all or only a limited number of independent variables could be studied. Second, exploration of possible sources of heterogeneity can be fallacious as the findings from these explorations can be subject to bias and confounding due to possible correlation that may exist between other measured or unmeasured study characteristic variables.^33^ A random effects parameter is usually included in the meta-regression models to explain any residual heterogeneity between studies that is not explained by the moderators included in the model.^25^ This leads to the following mixed effects meta-regression model:

(10) $${\hat{\theta }}_{i}=\beta_{1}+\alpha_{i}+b_{i}+\epsilon_{i}$$

where *α_i_*=*β*_2_*x_i_* when *x*_2*i*_ is a quantitative continuous covariate and *α_i_*=*β*_2_*x*_2*i*_+ … +*β_p_x_pi_* in the case of a factor with *p* levels and *p*−1 dummy variables *β*_2_*x*_2*i*_, *…, β_p_x_pi_*. As before, the sampling error is assumed to be normally distributed with mean 0 and known variance *ξ_i_*^2^ and *b_i_* ∼ *N*(0, *τ*^2^). When meta-regression models are fitted, while it is important to obtain an estimation for the model coefficients, it is also necessary to estimate how much variability exists between effect sizes. Subgroup analysis (partitioning of heterogeneity) could be used as an alternative to meta-regression. However, this approach is less powerful since stratification will lead to smaller subset of unpowered studies with poorer estimates of amount of heterogeneity in preclinical data.

#### Bayesian meta-regression

Note that estimation can be done, as in the random effects model, using either maximum likelihood or Bayesian inference. For the latter, vague prior distributions *N*(0,10^4^) are assigned to the regression parameters while an inverse gamma (0.001, 0.001) prior distribution is assigned to *τ*^2^.

## Results

We have applied the statistical methods described above to data from a systematic review conducted by the CAMARADES group.^7^ This review assessed the effect of treatment with IL-1RA versus control in animal models of ischaemic stroke. The primary outcome was reduction in infarct volume (76 observations) where NMD effect sizes were calculated. The secondary outcomes were improvement in neurobehavioural score (33 observations) and mortality (10 observations), which were calculated based on NMD and log OR, respectively. It is of interest to understand the impact of IL-1RA on these outcomes and to explain any heterogeneity between studies.

### Estimation of overall treatment effect

**Figure 1:**
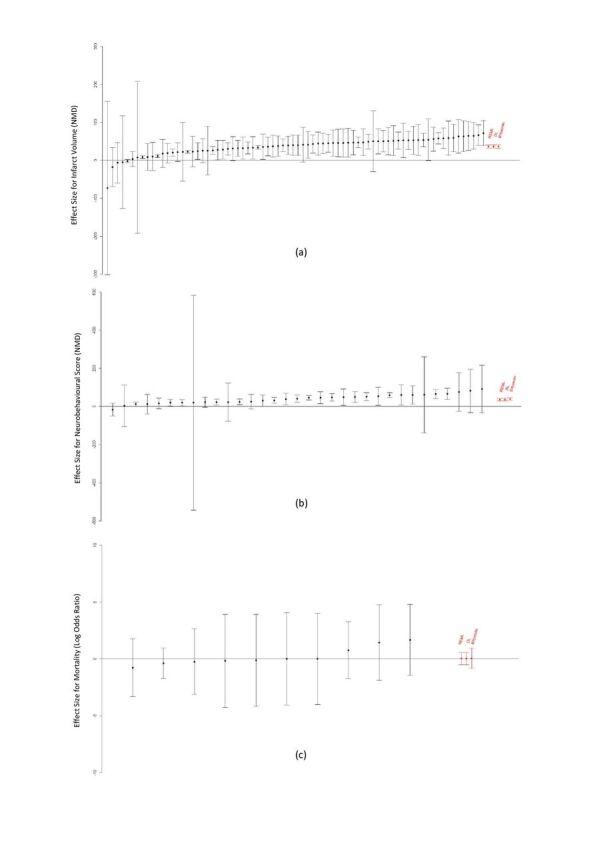
Effect of interleukin-1 receptor antagonist (IL-1RA) on (A) infarct volume (NMD), (B) neurobehavioural score (NMD), and (C) mortality (log OR) outcomes. Studies are ranked according to the effect size along with the vertical error bars representing 95% CI for individual effect size estimates. For each outcome, the overall random effects estimate of the treatment effect is also presented based on restricted maximum likelihood (REML), DerSimonian and Laird (DL) and Bayesian along with their 95% interval. NMD, normalised mean difference.

A random effects meta-analysis is performed using different estimation methods for the between-study variance. Results are presented in table 3. While the estimate of the overall treatment effect is similar for different estimation methods (see figure 1), considerable differences are observed for the estimation of the between-study heterogeneity. Infarct volume has large between-study variance, the DL method estimated larger between-study variance as compared with Bayesian and REML. Neurobehavioural score has moderate between-study variance (50%<I^2^<75%). In this case, the Bayesian method resulted in a higher *τ*^2^ estimate in comparison to REML and DL. For mortality data, the heterogeneity is very low (I^2^=0), and the Bayesian approach gives *τ*^2^ estimate larger than zero.

**Table 3:** Estimated overall effect sizes and corresponding CIs/credibility intervals for infarct volume (NMD), neurobehavioural score (NMD) and mortality data (log OR) using three different estimation methods for random effects meta-analysis of the effect of IL-1RA after stroke in animals

Outcome	Method	Estimate (95% CI)	*Q* statistic	$\hat{\tau }$(95% CI)	I^2^
Infarct volume	REML	36.2 (32.1 to 40.3)	423.4*	187.5 (72.5 to 234.0)	0.79
	DL	36.5 (32.4 to 40.6)	423.4*	236.9 (–)	0.82
	Bayesian	36.5 (32.4 to 40.6)		194.1 (113.6 to 314.2)	
Neurobehavioural score	REML	36.5 (32.4 to 40.6)	76.5*	208.0 (47.4 to 407.5)	0.59
	DL	35.9 (28.8 to 42.9)	76.5*	202.6 (–)	0.58
	Bayesian	38.9 (31.2 to 46.5)		317.7 (137.2 to 629.5)	
Mortality	REML	0.03 (−0.51 to 0.58)	2.87	0 (0.00 to 0.10)	0
	DL	0.03 (−0.51 to 0.58)	2.87	0 (–)	0
	Bayesian	0.04 (−0.82 to 0.93)		0.19 (0.001 to 1.38)	

*Indicates significance with α=0.05.

DL, DerSimonian and Laird; IL-1RA, nterleukin-1 receptor antagonist; NMD, normalised mean difference; REML, restricted maximum likelihood.

For infarct volume and neurobehavioural score outcomes, the estimates suggest an overall beneficial effect of IL-1RA treatment in animals with focal cerebral ischaemia in relation to the control group. There is significant between-study heterogeneity for infarct volume and neurobehavioural score outcomes (*Q=*423.4 and *Q=*76.5, p<0.01), unlike studies reporting mortality (*Q=*2.87, p=0.97).

The associated forest plots (see Appendix: https://osf.io/6usxq/) display the study-specific estimated difference in means between IL-1RA treated and control animals with 95% CIs based on the provided SEs of each study for both primary and secondary endpoints. The infarct volume and neurobehavioural score data contain highly heterogeneous effect sizes, in line with the different estimation techniques. Despite the fact that a large number of studies are expected to reduce the uncertainty of the overall treatment effect, the presence of heterogeneity may counteract this and lead to a net increase in uncertainty.^19^ Most of the individual studies report reduction of infarct volume in animals treated with IL-1RA, and an improvement in neurobehavioural measures. Overall estimates for the neurobehavioural score are similar, irrespective of the estimation technique used. However, the 95% intervals for the overall estimate are wider for the Bayesian approach compared with other estimation methods. Also, the results for the mortality data are similar for the overall mean of the log OR for different estimation methods. In this case, very little heterogeneity among studies is observed. It is important to realise that in random effects meta-analysis on mortality data using REML and DL, the heterogeneity is low but imprecisely estimated due to the small number of studies. Bayesian meta-analysis instead does not rely on large sample asymptotics. Despite the estimate for *τ*^2^ being a little higher than the frequentist meta-analysis approaches, the Bayesian approach results in a wider interval for the summary effect size since it appropriately takes into account all aspects of the parameterisation including uncertainties relating to the amount of data available.^34^

### Investigation of heterogeneity

For exploring heterogeneity using infarct volume data, we only considered the subset of studies where IL-1RA was administered in protein form. This was due to uncertainty around the timing and effective dose achieved in the experiments where IL-1RA was administered through transgenic or transfection manipulations. Thus, both study quality and design characteristics were analysed on 65 comparisons from 1055 animals.

To explain potential sources of heterogeneity for the primary outcome, infarct volume, we first performed univariate meta-regression on all study quality and study characteristic variables. As before, we compared three different *τ*^2^ estimation methods. Table 4 shows the estimates, CIs and heterogeneity measures obtained by adjusting for possible confounding features.

**Table 4:** Summary of selected univariate meta-regression results based on different estimation techniques using each study quality variable as a moderator to explain the study heterogeneity present in infarct volume data

Method	Variable	Estimate (SE/MC error)	95% Cl	P value	τ^2^	I^2^ (%)	R^2^ (%)
REML	No covariate	35.5 (2.35)	30.8 to 40.2	<0.0001	213.9	82.4	
	Blinded induction of ischaemia (intercept)	39.0 (2.76)	33.5 to 44.5	<0.0001	204.5	79.8	4.36
	Blinded induction of ischaemia (true)	−11.2 (4.88)	−21.0 to −1.46	0.03			
	Control of temperature (intercept)	38.2 (3.19)	31.8 to 44.5	<0.0001	219.1	81.0	0
	Control of temperature (false)	−5.67 (4.70)	−15.1 to 3.72	0.23			
DL	No covariate	35.7 (2.35)	31.0 to 40.4	<0.0001	235.2	83.7	–
	Blinded induction of ischaemia (intercept)	39.5 (2.76)	34.0 to 45.1	<0.0001	267.6	83.8	0
	Blinded induction of ischaemia (true)	−11.5 (4.88)	−21.2 to −1.73	0.02			
	Control of temperature (intercept)	38.6 (3.19)	32.2 to 45.0	<0.0001	268.4	83.9	0
	Control of temperature (false)	−6.00 (4.70)	−15.4 to 3.40	0.21			
Bayesian	No covariate	35.5 (0.03)	30.3 to 40.7	<0.0001	224.2		
	Blinded induction of ischaemia (intercept)	38.9 (0.06)	32.9 to 45.3	<0.0001	212.9		
	Blinded induction of ischaemia (true)	−11.1 (0.11)	−22.3 to −0.12	0.04			
	Control of temperature (intercept)	38.0 (0.07)	31.0 to 45.4	<0.0001	227.7		
	Control of temperature (false)	−5.58 (0.10)	−16.5 to 4.84	0.31			

P values are calculated based on Wald-type test statistics for frequentist approaches and using probability of direction for Bayesian approach as detailed in ref 39. Associated full tables can be found in the Appendix (https://osf.io/6usxq/) (Bayesian priors: $\beta ∼N(0,\;10^{4})$, $\tau^{2}∼IG(0.001,\;0.001)$).

DL, DerSimonian and Laird; MC, Monte Carlo; REML, restricted maximum likelihood.

A selection of the results is presented in table 4. The associated data and detailed analysis results are provided in the Appendix (https://osf.io/6usxq/). With respect to the impact of study quality, we found that infarct volume was reduced more in studies which did not report whether researchers were blinded to treatment allocation during the induction of ischaemia. Although the regression parameter is the same for all estimation methods, we observe large differences in terms of the impact on study heterogeneity. While we see that blinded induction reduces the study variability for the REML estimation from 213.9 to 204.5 and from 224.2 to 212.9 for the Bayesian estimation, we see an increase of *τ*^2^ from 235.2 to 267.6 for DL. This might have occurred due to the complex interplay between the heterogeneity estimate leading to overestimation of between-study heterogeneity in a meta-regression model and underestimation in the general random effects meta-analysis model.^19 22 25^

In general, parameter estimates are the same for different estimation methods. However, the Bayesian SDs are larger than those obtained using DL or REML. This is because Bayesian analysis allows for the uncertainty in its estimation of *τ*^2^. No other quality variables had a significant effect on heterogeneity, according to the univariate meta-regression models fitted.

With respect to the study characteristics as shown in table 5, the large amount of heterogeneity present in the data can be partially explained by the route of IL-1RA delivery. We noted that studies where IL-1RA was injected intracerebroventricularly reported significantly larger effect than studies where IL-1RA was administrated subcutaneously. In addition, studies using other administration methods reported significantly smaller effect than those reporting subcutaneous administration of the drug. Other study characteristic variables did not significantly contribute to heterogeneity. We also see that the REML method estimated smaller between-study variance value followed by Bayesian and DL. Although the performance of these estimation methods relies on how precisely the study weights are estimated, these differences are due to the different weighting schemes used by each method.^18^ For instance, while calculation of *τ*^2^ using DL considers only the inverse of the within-study variances, REML estimate makes use of both between-study and within-study variability in weighting. Although as heterogeneity increased, REML and DL methods converged to similar results. Based on our finding, and several studies both on real clinical data and simulations, REML is suggested to be a better alternative than DL as REML estimates lower between-study variances especially for continuous outcomes as in our case.^19 22 25 35 36^ Additionally, we observed that there was little difference in REML and DL versus the results obtained using Bayesian approach.

**Table 5:** Summary of the selected univariate meta-regression results based on different estimation techniques using each study characteristic variable as a moderator to explain the extra heterogeneity present in infarct volume data

Method	Variable	Estimate (SE/MC error)	95% Cl	P value	τ^2^	I^2^ (%)	R^2^ (%)
REML	No covariate	35.5 (2.35)	30.8 to 40.2	<0.0001	213.9	82.40	
	Time to outcome measure (intercept)	37.7 (2.75)	32.3 to 43.2	<0.0001	206.4	80.80	3.50
	Time to outcome measure	−0.03 (0.02)	−0.06 to 0.01	0.12			
	Route of drug delivery (intercept)	31.5 (3.27)	24.9 to 38.0	<0.0001	120.1	69.60	43.90
	Route of delivery (intracerebroventricular)	20.3 (5.54)	9.25 to 31.4	0.001			
	Route of drug delivery (other)	−14.6 (5.67)	−26.0 to −3.31	0.01			
	Route of drug delivery (intravenous)	5.79 (4.99)	−4.19 to 15.8	0.25			
	Dose (intercept)	38.0 (2.91)	32.2 to 43.8	<0.0001	221.9	81.70	0
	Dose	−0.02 (0.02)	−0.05 to 0.01	0.17			
DL	No covariate	35.7 (2.35)	31.0 to 40.4	<0.0001	235.2	83.70	
	Time to outcome measure (intercept)	38.0 (2.75)	32.5 to 43.5	<0.0001	253.3	83.80	0
	Time to outcome measure	−0.03 (0.02)	−0.06 to 0.01	0.13			
	Route of drug delivery (intercept)	32.4 (3.31)	25.8 to 39.1	<0.0001	177.8	77.20	24.40
	Route of delivery (intracerebroventricular)	19.3 (5.49)	8.28 to 30.3	0.001			
	Route of drug delivery (other)	−15.5 (5.99)	−27.4 to −3.50	0.01			
	Route of drug delivery (intravenous)	4.48 (5.02)	−5.57 to 14.5	0.38			
	Dose (intercept)	38.4 (2.91)	32.5 to 44.2	<0.0001	263.5	84.20	0
	Dose	−0.02 (0.02)	−0.05 to 0.01	0.15			
Bayesian	No covariate	35.5 (0.03)	30.3 to 40.7	<0.0001	224.2		
	Time to outcome measure (intercept)	37.6 (0.05)	31.7 to 37.6	<0.0001	215.2		
	Time to outcome measure	−0.03 (0.0004)	−0.07 to 0.01	0.14			
	Route of drug delivery (intercept)	31.5 (0.10)	24.4 to 39.3	<0.0001	127.7		
	Route of delivery (intracerebroventricular)	20.2 (0.15)	7.78 to 32.3	0.002			
	Route of drug delivery (other)	−14.6 (0.12)	−27.7 to −1.99	0.03			
	Route of drug delivery (intravenous)	5.85 (0.13)	−5.50 to 16.9	0.31			
	Dose (intercept)	37.9 (0.06)	31.7 to 44.7	<0.0001	232.9		
	Dose	−0.02 (0.0003)	−0.0 to 0.01	0.17			

P values are calculated based on Wald-type test statistics and mako. Associated full tables can be found in the Appendix (https://osf.io/6usxq/) ($\beta ∼N\left(0,10^{4}\right)$, $\tau^{2}∼IG\left(0.001,0.001\right)$).

DL, DerSimonian and Laird; MC, Monte Carlo; REML, restricted maximum likelihood.

It should be noted that although some variables may not produce a significant relationship with the change in infarct volume, it is possible that some of these are, in combination with other variables, important. Hence, we explored a multiple regression approach. Since we do not have a sufficient number of studies to fit an overall multivariable meta-regression model with all of the reporting of risk of bias and study characteristic variables, we performed a model building validation process, stepwise forward selection, to obtain the model that best explains the data. This was achieved by starting with a simple model, including only the two significant variables obtained from univariate analysis (blinded induction of ischaemia and route of drug delivery) and then adding other covariates one by one and recording the change in Akaike’s information criteria (AIC). When the model with the added predictor provided lower AIC than the previous model, indicating an improved fit of the model, the predictor was kept in the model as an important parameter. Although automatic stepwise model selection procedures exist, AIC has several benefits since it is asymptotically efficient and allows for simultaneous comparison of multiple nested and non-nested models.^37^ This led to a model with the following predictors: blinded induction of ischaemia, route of drug delivery, time to outcome measure, blinded assessment of outcome and dose. As a method to detect possible multicollinearity, we used variance inflation factor (VIF), which measures how much of the variances of the estimated regression coefficients are increased as compared with when the predictors do not have a linear relationship. According to the rule of thumb, a maximum VIF larger than 10 is considered as a serious indication of multicollinearity.^38^ When the VIF values were checked, we observed that blinded induction of ischaemia, route of drug delivery and blinded assessment of outcome had VIF values between 10 and 18, indicating existence of serious multicollinearity. Examination of the pairwise correlations showed high correlation between blinded assessment of outcome and route of delivery. Since both variables provided the same information, including only one in the model is sufficient and decreases the VIF values. In our case, we dropped each of these correlated variables in a stepwise manner and built the model again. We finally removed the variable which resulted in a model with a higher AIC value. Hence, the final model with predictors (blinded induction of ischaemia, route of drug delivery, time to outcome measure and dose) was selected as the best fitting model. The results from the model fitting are shown in table 6.

**Table 6:** Parameter estimates from multivariable meta-regression model using different estimation techniques for between-study heterogeneity (Bayesian priors: $\beta ∼N\left(0,10^{4}\right)$, $\tau^{2}∼IG\left(0.001,\;0.001\right)$)

Method	Variable	Estimate (SE/MC error)	95% Cl
REML	$\beta_{1}$(Intercept)	42.9 (5.53)*	31.8 to 54.0
	$\beta_{2}$(Blinded induction of ischaemia: true)	−11.5 (7.03)	−25.5 to 2.60
	$\beta_{3}$(Route of delivery: intracerebroventricular)	10.1 (6.80)	−3.55 to 23.7
	$\beta_{4}$(Route of delivery: intravenous)	−2.85 (6.10)	−15.1 to 9.37
	$\beta_{5}$(Route of delivery: other)	−24.4 (6.66)*	−37.8 to −11.1
	$\beta_{6}$(Time to outcome measure)	−0.03 (0.02)	−0.07 to 0.01
	$\beta_{7}$(Dose)	0.01 (0.02)	−0.03 to 0.05
DL	$\beta_{1}$(Intercept)	43.6 (5.47)*	32.6 to 54.5
	$\beta_{2}$(Blinded induction of ischaemia: true)	−10.9 (6.92)	−24.7 to 2.97
	$\beta_{3}$(Route of delivery: intracerebroventricular)	9.28 (6.69)	−4.12 to 22.7
	$\beta_{4}$(Route of delivery: intravenous)	−3.71 (6.04)	−15.8 to 8.39
	$\beta_{5}$(Route of delivery: other)	−24.7 (6.84)*	−38.4 to −11.0
	$\beta_{6}$(Time to outcome measure)	−0.03 (0.02)	−0.07 to 0.01
	$\beta_{7}$(Dose)	0.01 (0.02)	−0.03 to 0.04
Bayesian	$\beta_{1}$(Intercept)	39.0 (0.22)*	27.4 to 50.2
	$\beta_{2}$(Blinded induction of ischaemia: true)	−13.7 (0.34)	−31.1 to 3.83
	$\beta_{3}$(Route of delivery: intracerebroventricular)	12.6 (0.23)	−1.67 to 27.2
	$\beta_{4}$(Route of delivery: intravenous)	0.05 (0.22)	−13.1 to 12.9
	$\beta_{5}$(Route of delivery: other)	−20.9 (0.21)*	−35.3 to −6.25
	$\beta_{6}$(Time to outcome measure)	−0.003 (0.001)	−0.05 to 0.05
	$\beta_{7}$(Dose)	0.01 (0.001)	−0.03 to 0.06

*Represents significant effect of a predictor at an alpha level of 0.05.

DL, DerSimonian and Laird; MC, Monte Carlo; REML, restricted maximum likelihood.

The inclusion of these variables to improve model fit led to a substantial drop in between-study variance with *τ*^2^_REML_=109.3, *τ*^2^_DL_=168.5 and *τ*^2^_FB_=123.6 in comparison to the heterogeneity variance observed with the random effects meta-analysis model or univariate meta-regressions. Similar to univariate analysis, there is still moderate to high heterogeneity present in the data (as measured by I^2^), where REML estimated lower *τ*^2^ as compared with Bayesian and DL (I^2^_REML_=68.3%, R^2^_REML_=50.2%, I^2^_DL_=76.9%, R^2^_DL_=28.8%). After correcting for time to outcome, dose and blinded induction, route of delivery is a significant source of heterogeneity, indicating that studies where IL-1RA was delivered subcutaneously showed less reduction in infarct volume than when IL-1RA was delivered in other ways (table 6). Note, however, that the estimates of the reported variables are not precise with wide 95% CI and credibility intervals. As before, the Bayesian analysis yielded a slightly wider 95% range than those obtained using REML since the *τ*^2^ estimate was relatively larger in Bayesian analysis. There can be considerable sensitivity to the prior distribution which may impact our results and conclusions. Hence, to check for the robustness of our choice of the Bayesian prior distribution for *τ*^2^, we conducted a sensitivity analysis assuming a different prior distribution. We altered the inverse gamma (0.001, 0.001) prior assumed for between-study heterogeneity by a positive half-normal (0, 10) and a positive half-normal (0, 100) prior distributions and examined if our posterior mean parameter estimates changed substantially.^39^ We observed the *τ*^2^ estimates obtained to be equal to 180.3 using both half-normal priors with correspondingly smaller *β* estimates and Monte Carlo errors. This illustrates that although the prior is aimed to be non-informative, it still causes some change in the obtained results due to the limited amount of data at hand.

## Discussion

Analyses that explore whether certain predictors can explain the heterogeneity in meta-analysis outcomes are becoming more common in preclinical fields. This informs a better understanding of the meta-analysis results by using the information provided in the studies. Univariate meta-regression is the conventionally used method in practice. In this paper, we investigated the use of univariate and multivariable meta-regressions, while exploring different between-study variability estimation techniques. As can be seen in the presented results, the amount of heterogeneity explained has an impact on the parameter estimates and SEs.

While in a meta-analysis setting without any covariates, it is more common to use the DL method for between-study heterogeneity estimation as it is non-iterative, calculations become complex when several covariates are introduced.^33^ Simulation studies suggested that DL underestimates between-study variance when the underlying level of variability is large.^22 36^ Also, when the outcome of interest in meta-analysis is continuous, the DL estimator is reported to provide a relatively comparable mean squared error and higher variance as compared with the REML estimator.^19^ The DL estimator is negatively biased when there is moderate to high between-study heterogeneity. This bias in DL occurs due to the false assumption that within-study variabilities, which are used for weight calculations, are known, with no associated uncertainty; when in practice they are estimated. As an alternative, we used REML as it excludes the summary effect parameter in its estimation of *τ*^2^ (unlike to DL), and is expected to reduce bias. Yet REML also has its limitations, such that the likelihood ratio test cannot be used to compare models with fixed effects parameters, it involves an iterative procedure and, although rare, is not always guaranteed to reach convergence.^40^ Hence, we exemplified the use of the Bayesian method as a between-study heterogeneity estimator, since it already accounts for uncertainty of the parameters by simultaneously estimating the *τ*^2^ along with other parameters. It is also important to note that while all other mentioned methods for heterogeneity estimation assume within-study variances are known, only the Bayesian approach does not necessitate this assumption and does not rely on large sample asymptotic normality, as assumed in maximum likelihood methods.

Additionally, the methods described here assume that the treatment effects are normally distributed. This is usually just an approximation which gets more correct as the within-study sample sizes get larger.^41^ However, due to small sample of animals and publication bias, this might not always be the case in meta-analyses of preclinical data. Normality of the data is also assumed in the Bayesian heterogeneity estimation method; however, other distributions can be easily assumed and implemented.^42^

Unexplained sources of heterogeneity can be identified by adding study-level predictors to random effects meta-regression. In most publications, investigation of the relationship between the effect of a treatment and study-level predictors is evaluated using univariate meta-regression due to the limited number of studies included in the data. However, this limitation can be handled using a model building exercise to obtain the best fitting multiple regression model to the observed data, rather than fitting one model with all the covariates included. Unlike the univariate meta-regression approach, the multiple meta-regression approach provides the ability to detect the relative impact of multiple covariates on the dependent variable. Multivariable meta-regression explains substantially more heterogeneity than univariate meta-regression while also leading to more generalisable results.

## Conclusion

In this study, we have illustrated the application of most common heterogeneity estimation methods in preclinical setting and presented our findings on key differences between these methods. Based on several applications and simulation studies on clinical data, REML and Bayesian heterogeneity estimation methods are recommended as they both show promise for meta-analysis when there is high heterogeneity; in comparison, DL is often biased.^19 22 25 36 43^ REML is advised as it is already commonly known and easily implemented in most statistical software. Although similar to REML, the non-iterative Bayesian approach has additional several advantages, including that it does not assume known within-study variances, distributions other than normal can be easily assumed for the random effects and, when available, known priors can be integrated to strengthen our conclusions. In conclusion, our findings suggest that despite substantial differences in data structure, and in particular the preponderance of heterogeneity being between studies (preclinical) rather than within studies (clinical), the consensus that REML and Bayesian heterogeneity estimation have advantages over DL heterogeneity estimation holds for systematic reviews of preclinical data. For quantifying and explaining variability between studies, multivariable meta-regression can be considered using a model building process. This method is valuable to investigate the relative importance of multiple study design and characteristic variables simultaneously and can explain more of the variability in data than univariate meta-regression.
